# Assessment of histopathology and cytology request form documentation quality using six Sigma and Pareto analysis in Benghazi, Libya

**DOI:** 10.1186/s13000-025-01740-0

**Published:** 2025-12-17

**Authors:** Hussien Hamid, Hamza Naas, Mohamed A. Alshaqabi, Moutaz F. Gebril, Nabeia A. Gheryani, Abdel Alhakem Alhabone, Mohamed H. S. Ahmida, Abdulla M. Elmansoury, Mohamed Najah

**Affiliations:** 1 Clinical Laboratory Sciences Program, Libyan International University, Benghazi, Libya; 2 Diagnostic Center, Libyan International University, Benghazi, Libya; 3 Department of Pathology, Faculty of medicine, University of Benghazi, Benghazi, Libya; 4 AL-Saleem Medical Laboratory, Benghazi, Libya; 5 Basic Medical Sciences Program, Libyan International University, Benghazi, Libya

**Keywords:** Histopathology, Pre-analytical errors, Six sigma, Pareto analysis, Laboratory quality, Request form documentation

## Abstract

**Background:**

Histopathology and cytology request forms are pivotal in the pre-analytical phase of laboratory testing, where incomplete or erroneous documentation on these forms can compromise the entire testing process. This study aimed to assess the documentation quality and process performance of histopathology and cytology request forms using Six Sigma and Pareto analysis in three private laboratories in Benghazi, Libya.

**Methods:**

A retrospective cross-sectional study was conducted on 1,181 request forms collected from February to April 2025. A structured checklist encompassing five documentation domains and 15 quality indicators based on WHO guidelines was used to assess form completeness. Six Sigma metrics including Defects per Unit (DPU), Defects per Million Opportunities (DPMO), Sigma level, and Yield (%), along with Pareto analysis, were applied to evaluate and prioritize quality deficiencies.

**Results:**

None of the evaluated request forms achieved full compliance with documentation standards. The overall process performance was unacceptable, with a Sigma level of 1.707 and a yield of 58.22%. Pareto analysis revealed that approximately 80% of documentation errors originated from three key domains: requesting clinician details, personal information, and clinical information. The requesting clinician details domain was the most deficient, with a Sigma level of 1.438 and a yield of 47.5%. The personal information domain followed, with a Sigma level of 1.867 and a yield of 64.31%. The clinical information domain showed a Sigma level of 1.263 and a yield of 40.6%. In contrast, the specimen details domain exhibited relatively better performance, with a Sigma level of 2.574 and a yield of 85.86%.

**Conclusions:**

Six Sigma and Pareto analysis were applied to identify critical deficiencies in documentation practices during the pre-analytical phase of histopathology services in Libya. The results highlight an urgent need to implement standardized staff training protocols, redesign request forms with mandatory fields, enforce accountability mechanisms, and establish robust quality monitoring systems. Low-cost tools—such as Excel-based compliance trackers and manual logbooks—can serve as effective interim solutions to enhance documentation compliance and support continuous quality improvement in resource-limited settings.

**Supplementary Information:**

The online version contains supplementary material available at 10.1186/s13000-025-01740-0.

## Highlights

No request forms met full documentation standards in all required domains.

The overall process showed poor performance with a Sigma level of only 1.707.

80% of errors came from clinician, personal, and clinical information fields.

The clinician details domain had the highest defect rate and lowest process yield.

Specimen details domain showed comparatively better documentation quality.

**Supplementary Information:**

The online version contains supplementary material available at 10.1186/s13000-025-01740-0.

## Introduction

Anatomic pathology, encompassing histopathology, cytopathology, and autopsy examination, is fundamental to disease diagnosis and clinical decision-making. Histopathology serves as the gold standard for evaluating surgical specimens. Advances in immunohistochemistry, molecular diagnostics, and morphometric analysis enhance diagnostic accuracy, particularly in oncology [1, 2]. These advancements depend on the accuracy of the total testing process (TTP). The pre-analytical phase is the most error-prone, accounting for up to 70% of laboratory-related errors, and directly impacts diagnostic reliability and patient safety [2–4].

In histopathology, the pre-analytical phase comprises procedures from specimen receipt to slide preparation. Errors during this phase are frequent [5]. Common errors include specimen mislabeling, inadequate fixation, and incomplete clinical information, often due to deficient request forms. One study found that 92.9% of nonconformities in tissue processing were related to pre-analytical errors, highlighting their prevalence [4].

Addressing these vulnerabilities is emphasized in International Organization for Standardization Technical Specifications such as ISO/TS 22367:2008, which recommends that clinical laboratories identify high-risk processes, monitor safety incidents, and implement corrective actions [6].

Accurate histopathology request forms are essential for effective laboratory functioning. These forms should include critical patient identifiers, detailed clinical history, surgical notes, specimen description, differential diagnoses, and referring physician information [7]. Deficient clinical documentation on request forms is a persistent challenge in histopathology, directly limiting pathological correlation and compromising diagnostic precision, as confirmed by international incident reports [8–10]. An Iranian study of 2,040 histopathology request forms found that none were fully complete, while basic identifiers were present in over 90% of cases, critical clinical information such as specimen description was included in only 12.5% [11]. In Egypt, 21.7% of forms lacked the patient’s name and 16.4% were missing clinical information [12]. Research from Sudan reported that 48.3% of forms lacked essential clinical history, 29.5% were missing differential diagnoses, 17.4% of specimens were submitted without formalin, and 5.3% arrived unlabeled [13].

Six Sigma is a data-driven quality management methodology used to promote continuous process improvement in clinical laboratories [14, 15]. It evaluates process performance based on defect rates, where higher sigma values correspond to lower error rates. A Six Sigma performance level (≥ 6σ) equates to fewer than 3.4 defects per million opportunities (DPMO). According to ISO 15189:2022, quality indicators such as yield%, defects%, DPMO, and Six Sigma metrics serve as performance measures to assess how well laboratory processes meet quality standards [16]. A Nigerian study applied Six Sigma metrics to 17 pre-analytical quality indicators in a chemical pathology laboratory, revealing that 70.6% demonstrated unacceptable performance [17].

While research has explored laboratory errors across the Total Testing Process, growing attention has focused on the pre-analytical and post-analytical stages, which are critical to diagnostic accuracy but often occur outside direct laboratory control [10]. Although numerous studies have examined pre-analytical errors in histopathology request forms, most have relied on basic descriptive statistics, with limited use of structured quality improvement tools such as Six Sigma and Pareto analysis.

Pathology services in Africa face challenges including personnel shortages, insufficient infrastructure, and limited funding [18]. Libya’s health information system is underdeveloped, with paper-based records and non-interoperable systems hindering patient care and planning [19].

To our knowledge, no prior studies in this context have applied structured quality improvement methodologies like Six Sigma and Pareto analysis. The findings are expected to inform policy decisions, promote regulatory reform, and guide the development of targeted training programs to strengthen laboratory systems in Libya and comparable settings.

## Methods

### Study design and setting

This study employed a retrospective, cross-sectional design to evaluate the quality of documentation in histopathology and cytology request forms. The study was conducted in three private histopathology laboratories in Benghazi, Libya, selected through purposive sampling. These laboratories were chosen for the following reasons: (1) they are leading private sector providers in the city, processing high specimen volumes that reflect routine clinical practice; (2) their operational structure is representative of private laboratory services in the region, allowing for findings that are relevant to this specific and under-researched sector; and (3) they granted administrative approval and provided access to their archived records.

### Sampling strategy and data collection

Data were collected from all archived request forms over a three-month period (February–April 2025). During this period, approximately 1,470 forms were archived across the three laboratories, constituting the study’s sampling frame. We employed a consecutive sampling approach, retrospectively reviewing all forms that met the inclusion criteria (histopathology and cytology requests with complete archival records). This yielded a final sample of 1,181 eligible forms from three anonymous laboratories (323 from Laboratory A, 561 from Laboratory B, and 297 from Laboratory C). Forms related to other laboratory services or identified as duplicate entries were excluded.

#### Sampling limitation

While the consecutive sampling of all eligible forms within a defined timeframe strengthens internal validity, the restriction to three private laboratories in a single city limits the generalizability of the findings to public hospitals or other geographical regions in Libya.

### Data collection tool and variables

A structured data extraction checklist was developed based on the 2019 World Health Organization (WHO) guidance on standardized histopathology and cytology request forms [20] and relevant clauses from ISO 15189:2022 pertaining to pre-examination documentation. The tool was designed to assess 15 quality indicators distributed across five pre-analytical domains, which were selected as they represent the minimum essential elements for accurate specimen identification, clinician-pathologist communication, and process traceability [7, 10].

The complete checklist is provided as a Supplementary File (Annex 1). In brief, the domains and their indicators were:

- *Patient identification:* Full name (≥ 3 components), date of birth/age, gender, contact information.
- *Clinical information*: Provisional diagnosis/suspicion, relevant medical history.
- *Specimen details*: Hospital name, specimen type, anatomical site, date/time of collection.
- *Requesting clinician Information*: Name, specialty, contact details, signature.
- *Ancillary investigations:* Request for special stains, immunohistochemistry, or molecular tests.

### Tool validation and data extraction

The checklist was pilot-tested on 50 randomly selected forms to ensure clarity and feasibility. Inter-rater reliability, assessed using Cohen’s kappa coefficient, was excellent (κ = 0.85). Any discrepancies between assessors were resolved through consensus discussion prior to the full data collection. Each variable was operationally defined; documentation was scored as “mentioned” only if the information was completely and legibly recorded, and “missing” if absent or incomplete.

#### Statistical analysis

Descriptive statistics, including frequencies and percentages, were generated using Statistical Package for the Social Sciences (SPSS) version 25.0. To evaluate the quality performance of histopathology and cytology request forms, Six Sigma performance indicators—Defects per Unit (DPU), Defects per Million Opportunities (DPMO), sigma levels, and process yield (i.e., the percentage of defect-free forms per domain)—were calculated for each documentation domain and for the overall form performance using the Benchmark Six Sigma^®^ online calculator platform. The Sigma level was calculated using the standard Six Sigma formula:

DPMO (Defects per Million Opportunities) = (Total defects ÷ [Number of units × Opportunities per unit]) × 1,000,000 [21]. The resulting DPMO value was then compared with the standard Six Sigma conversion table (Table 1) to determine the corresponding Sigma level and yeild% [22].

**Table 1:** Standard six Sigma performance scale showing corresponding DPMO and yield values

Sigma Level	Defects per Million Opportunities (DPMO)	Yield (%)
1σ	698,000	31%
2σ	308,537	69.1%
3σ	66,807	99.3%
4σ	6,210	99.4%
5σ	233	99.98%
6σ	3.4	99.9997%

Reproduced from 6Sigma.com [22]. Open access: Permission granted

Performance levels were then interpreted by referencing the corresponding sigma values on the Sigma performance scale presented in (Table 2) [16]. A Pareto chart, a quality improvement tool used to identify the most frequent error categories, was constructed using Excel software.

**Table 2:** Interpretation of sigma metrics

Sigma value	Indication
σ value ≥ 6	World-class performance
σ value ≥ 5	Excellent performance
σ value ≥ 4	Good Performance
σ value ≥ 3	Marginal Performance
σ value ≥ 2	Poor Performance
σ value < 2	Unacceptable performance

Reproduced from Hamid H et al. [16]. Open access: Permission granted

### Ethical considerations

This study was approved by the Libyan International University Research Ethics Committee (Reference No. BMS-2025-00026; Project ID: MHS-12-G-00420), in accordance with the Declaration of Helsinki. The study involved retrospective review of archived histopathology and cytology request forms. Although the forms contained patient-identifiable information such as names and age, no patient contact occurred, and no personal data were recorded or used in the analysis. Given the nature of the study, the Research Ethics Committee waived individual informed consent and granted permission to access the data. Administrative approval was also obtained from the participating laboratories.

## Results

None of the 1,181 evaluated histopathology and cytology request forms was fully complete. The overall documentation process was suboptimal, with a Sigma level of 1.707 and a defect-free yield of 58.22% (Table 3).

**Table 3:** Completeness of histopathology and cytology request forms across five core domains in private laboratories (N = 1,181)

Request Form Domains	Variables	Response Category	Frequency (*n*)	Percent (%)
Personal Information	Full Name (≥ Three components)	Missing	154	13
		Mentioned	1027	87
	Date of Birth/Age	Missing	136	11.5
		Mentioned	1045	88.5
	Gender	Missing	523	44.3
		Mentioned	658	55.7
	Contact Info (Address, Phone)	Missing	873	73.9
		Mentioned	308	26.1
Clinical Information	Clinical Diagnosis/Suspicion	Missing	608	51.5
		Mentioned	573	48.5
	Relevant Medical History	Missing	794	67.2
		Mentioned	387	32.8
Specimen Details	Hospital Name	Missing	85	7.2
		Mentioned	1096	92.8
	Type of Specimen	Missing	168	14.2
		Mentioned	1013	85.8
	Site of Origin	Missing	213	18
		Mentioned	968	82
	Date & Time of Collection	Missing	202	17.1
		Mentioned	979	82.9
Requesting Clinician\Surgeon Information	Name	Missing	227	19.2
		Mentioned	954	80.8
	Specialty	Missing	812	68.8
		Mentioned	369	31.2
	Contact Information	Missing	1166	98.7
		Mentioned	15	1.3
	Signature	Missing	274	23.2
		Mentioned	907	76.8
Ancillary Investigations	Special Stains/IHC/Molecular Tests	Missing	1167	98.8
		Mentioned	14	1.2

Substantial variability was observed across domains (Table 4). The Specimen Details domain demonstrated the highest, though still poor, performance (Sigma level: 2.574; yield: 85.86%). In contrast, the Clinical Information and Requesting Clinician Information domains showed critical deficiencies, with low Sigma levels (1.263 and 1.438, respectively) and yields below 50%. The Ancillary Investigations domain exhibited near-complete non-compliance, with a negative Sigma level (−0.762) and a yield of only 1.19%.

**Table 4:** Six sigma metrics of documentation in histopathology and cytology request forms (N = 1,181)

Request Form Domains	Number of Non-conformities	Opportunities Per Unit	Defect per Units (DPU)	Defects Per Million Opportunities (DPMO)	Yield%	Sigma Level (within)		Performance Level
Personal Data	1,686	4	1.428	356900.931	64.31%	1.867σ	Unaccepted	
Clinical Information	1,402	2	1.2	593564.776	40.644%	1.263σ	Unaccepted	
Specimen Details	668	4	0.566	141405.588	85.859%	2.574σ	Poor	
Requesting Clinician\Surgeon Information	2,479	4	2.099	524767.146	47.523%	1.438σ	Unaccepted	
Ancillary investigations	1,167	1	0.988	988145.639	1.185%	−0.762σ	Unaccepted	
Overall performance	7,402	15	6.268	417837.99	58.216%	1.707σ	Unaccepted	

**Fig. 1:**
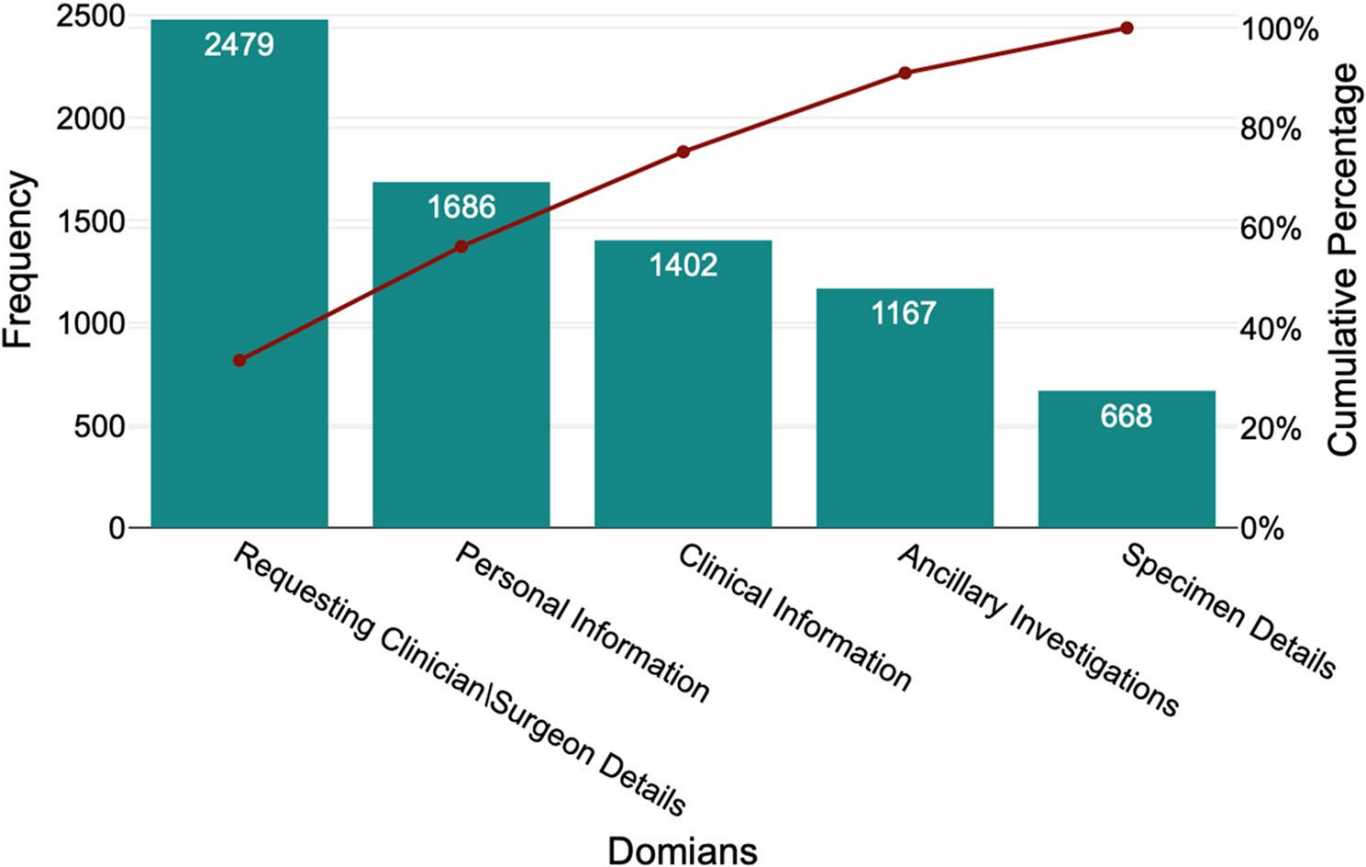
Pareto chart showing frequency and cumulative percentage of documentation errors across five domains, highlighting major deficiencies per the 80/20 principle

Pareto analysis (Fig. 1) revealed that documentation errors were highly concentrated in just three domains: Requesting Clinician/Surgeon Information, Personal Information, and Clinical Information, which collectively accounted for approximately 80% of all errors.

## Discussion

This study evaluates the quality of histopathology and cytology request forms in Libyan private laboratories using Six Sigma and Pareto analysis. The finding that none of the 1,181 request forms was fully complete aligns with similar studies in low-resource settings, such as Nigeria [23], and underscores a systemic challenge in the pre-analytical phase.

The overall Sigma level of 1.707 indicates an unacceptably high error rate, below the benchmark for clinical processes [7]. This performance is notably poorer than that reported in a Nigerian chemical pathology laboratory, where 17.6% of indicators achieved good performance [17], and contrasts with higher compliance rates documented in Pakistan [24]. This global variability highlights the influence of local systems and resources on quality outcomes. Pareto analysis identified that approximately 80% of errors were concentrated in three domains (clinician details, personal identifiers, and clinical data) providing a clear target for prioritizing improvements to request forms.

The poor performance in the Clinical Information domain (σ = 1.263) is concerning because missing provisional diagnoses or medical history impedes pathological correlation [12, 24]. This aligns with European molecular pathology data, where most pre-analytical incidents were linked to request form deficiencies [10]. Similarly, the lack of clinician specialty and contact information (σ = 1.438) creates a communication barrier that can lead to diagnostic delays and errors, a recognized risk in patient safety frameworks [25].

While the Specimen Details domain performed best (σ = 2.574), it remains below the acceptable threshold, a finding consistent with previous studies [11]. This suggests that even the more routinely documented elements require reinforcement. The near-total absence of requests for ancillary tests (σ = −0.762) mirrors underutilization reported elsewhere [26] and represents a major gap in diagnostic capability, potentially affecting cancer management. These deficiencies represent significant patient safety risks. They reflect a broader, global issue with pre-analytical quality, as noted in similar studies [8, 26]. In the Libyan context, these failures likely stem from a combination of factors, including a lack of standardized procedures, insufficient training, and the absence of an electronic ordering system that could enforce data completeness.

### Implications and recommendations

Our findings support the need for targeted interventions. Priority should be given to the top error-prone domains identified by the Pareto chart. Initial, low-cost steps include:

- *Form redesign*: Introducing a single-page, standardized request form with mandatory fields for critical clinical data and clinician contact information.
- *Audit and feedback*: Implementing regular audits of form completeness with feedback to clinicians.
- *Structured training:* Brief, focused training sessions for clinical staff on the impact of complete documentation on diagnostic accuracy.

While the integration of Laboratory Information Systems (LIS) or Electronic Health Records (EHR) is a long-term goal, these manual, systemic interventions can yield significant immediate improvement in data quality and, consequently, patient care.

## Conclusion

This study identifies significant deficiencies in histopathology and cytology request form documentation, with Six Sigma metrics indicating poor performance in the pre-analytical phase. The application of Six Sigma and Pareto analysis precisely quantified this deficit and identified clinician details, patient identifiers, and clinical information as the primary error sources. These findings call for a systematic response, including standardized forms, staff training, and audit cycles. Such evidence-based interventions are essential to strengthen diagnostic accuracy and laboratory efficiency.

### Study limitations

This study has several limitations that should be acknowledged. First, it was limited to private laboratories within a single city (Benghazi), which may restrict the external validity and generalizability of the findings to public healthcare settings or other regions of Libya with different administrative structures and resource levels. Second, the retrospective design, which relied solely on the review of archived request forms, limits the ability to infer causality or assess the downstream clinical impact of documentation errors on diagnostic accuracy and patient outcomes. Third, the use of convenience sampling may have introduced selection bias, potentially over- or underestimating the true prevalence of documentation deficiencies. Furthermore, as the data were extracted from existing records, the accuracy of the findings depends on the completeness and integrity of the archived forms, and there is a potential for misclassification of documented data due to illegible handwriting or subjective interpretation of entries. Finally, while Six Sigma and Pareto analyses provided valuable quantitative assessments of process performance, the absence of complementary qualitative methods—such as staff interviews or workflow observations—precluded identification of behavioral, procedural, or system-level root causes underlying non-compliance.

Future studies should aim to validate these findings through multicenter investigations that include both private and public laboratories across diverse geographic regions. Incorporating mixed-method approaches, such as qualitative interviews or root cause analyses, could provide deeper insights into human and systemic factors contributing to documentation errors. Longitudinal and interventional studies are also recommended to assess the effectiveness of targeted training, digital request form systems, and quality improvement interventions on reducing pre-analytical non-conformities and enhancing laboratory data validity.

## Supplementary Information

Supplementary Material 1

Supplementary Material 2

## Data Availability

Additional data and supporting materials are available from the corresponding author upon reasonable request.
