# Supplementary material for: Assessment of histopathology and cytology request form documentation quality using six Sigma and Pareto analysis in Benghazi, Libya
Source: Diagn Pathol. 2025 Dec 17;21:6. doi: 10.1186/s13000-025-01740-0 (PMC12821826; doi:10.1186/s13000-025-01740-0)
Supplement: Supplementary file 1 — Supplementary Material 1 [file 13000_2025_1740_MOESM1_ESM.docx]

**Table 1: Interpretation of sigma metrics.**

| **Sigma value** | **Indication** |
| --- | --- |
| σ value ≥ 6 | World-class performance |
| σ value ≥ 5 | Excellent performance |
| σ value ≥ 4 | Good Performance |
| σ value ≥ 3 | Marginal Performance |
| σ value ≥ 2 | Poor Performance |
| σ value < 2 | Unacceptable performance |
| **Table 1.** Reproduced from Hamid H et al. [17]. Open access: Permission granted. | |

**Table 2: Completeness of Histopathology and Cytology Request Forms Across Five Core Domains in Private Laboratories (N = 1,181): A Structured Audit Based on CAP Guidelines**

| **Request Form Domains** | **Variables** | **Response Category** | **Frequency (n)** | **Percent (%)** |
| --- | --- | --- | --- | --- |
| Personal Information | Full Name (≥ Triple) | Missing | 154 | 13 |
|  |  | Mentioned | 1027 | 87 |
|  | Date of Birth / Age | Missing | 136 | 11.5 |
|  |  | Mentioned | 1045 | 88.5 |
|  | Gender | Missing | 523 | 44.3 |
|  |  | Mentioned | 658 | 55.7 |
|  | Contact Info (Address, Phone) | Missing | 873 | 73.9 |
|  |  | Mentioned | 308 | 26.1 |
| Clinical Information | Clinical Diagnosis / Suspicion | Missing | 608 | 51.5 |
|  |  | Mentioned | 573 | 48.5 |
|  | Relevant Medical History | Missing | 794 | 67.2 |
|  |  | Mentioned | 387 | 32.8 |
| Specimen Details | Hospital Name | Missing | 85 | 7.2 |
|  |  | Mentioned | 1096 | 92.8 |
|  | Type of Specimen | Missing | 168 | 14.2 |
|  |  | Mentioned | 1013 | 85.8 |
|  | Site of Origin | Missing | 213 | 18 |
|  |  | Mentioned | 968 | 82 |
|  | Date & Time of Collection | Missing | 202 | 17.1 |
|  |  | Mentioned | 979 | 82.9 |
| Requesting Clinician\Surgeon Information | Name | Missing | 227 | 19.2 |
|  |  | Mentioned | 954 | 80.8 |
|  | Specialty | Missing | 812 | 68.8 |
|  |  | Mentioned | 369 | 31.2 |
|  | Contact Information | Missing | 1166 | 98.7 |
|  |  | Mentioned | 15 | 1.3 |
|  | Signature | Missing | 274 | 23.2 |
|  |  | Mentioned | 907 | 76.8 |
| Ancillary Investigations | Special Stains / IHC / Molecular Tests | Missing | 1167 | 98.8 |
|  |  | Mentioned | 14 | 1.2 |

**Table 3: Six Sigma Performance Metrics Across Key Domains of Histopathology Request Forms: Analysis of DPU, DPMO, Yield, and Sigma Levels (N = 1,181)**

| **Request Form Domains** | **Number of Non-conformities** | **Opportunities Per Unit** | **Defect per Units**  **(DPU)** | **Defects Per Million Opportunities (DPMO)** | **Yield%** | **Sigma Level**  **(within)** | | **Comment** |
| --- | --- | --- | --- | --- | --- | --- | --- | --- |
| Personal Data | 1,686 | 4 | 1.428 | 356900.931 | 64.31% | 1.867σ | Unaccepted | |
| Clinical Information | 1,402 | 2 | 1.2 | 593564.776 | 40.644% | 1.263σ | Unaccepted | |
| Specimen Details | 668 | 4 | 0.566 | 141405.588 | 85.859% | 2.574σ | Poor | |
| Requesting Clinician\Surgeon Information | 2,479 | 4 | 2.099 | 524767.146 | 47.523% | 1.438σ | Unaccepted | |
| Ancillary investigations | 1,167 | 1 | 0.988 | 988145.639 | 1.185% | -0.762σ | Unaccepted | |
| **Total** | **7,402** | **15** | **6.268** | **417837.99** | **58.216%** | **1.707σ** | Unaccepted | |


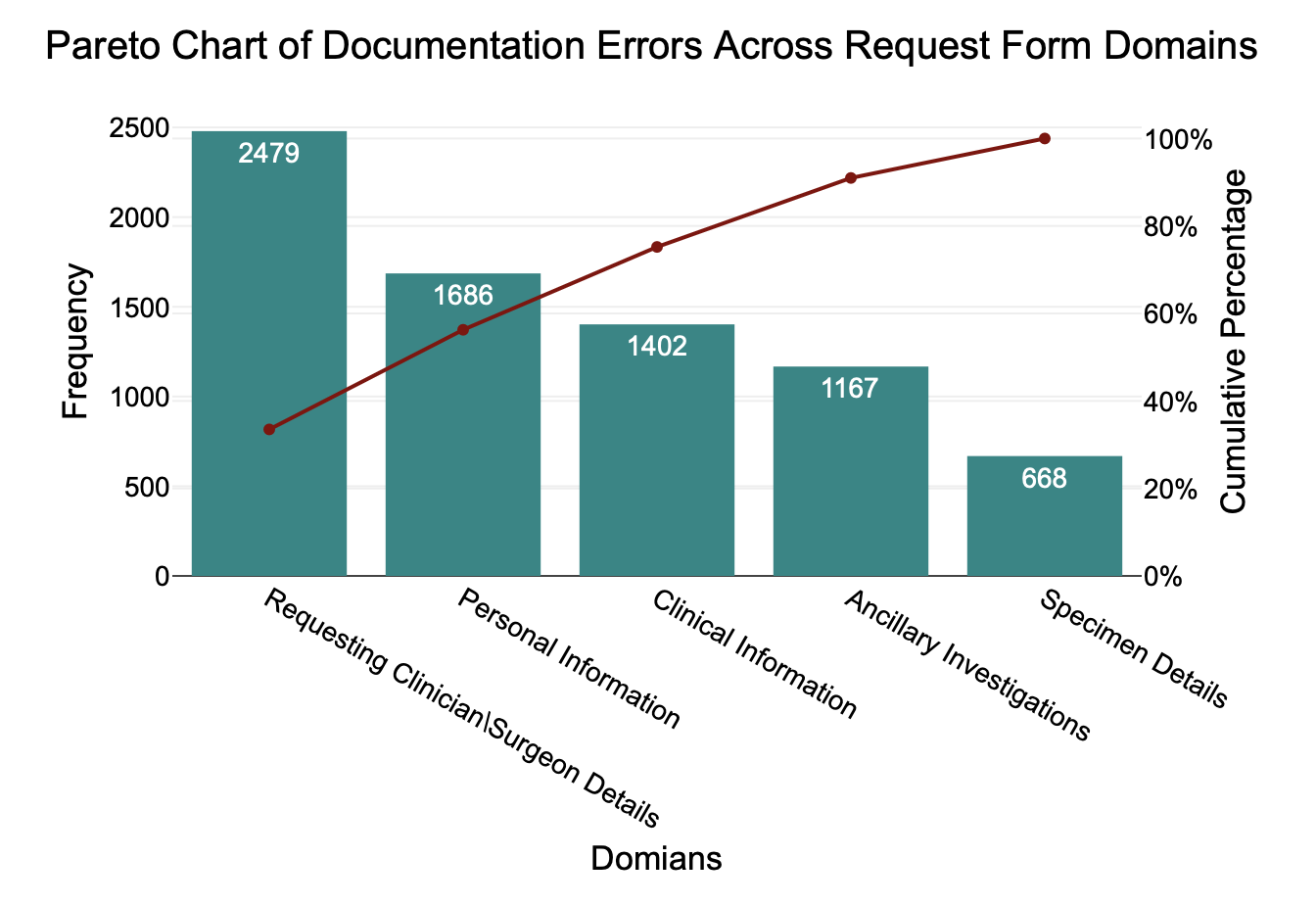
 **Figure 1: Pareto Chart of Documentation Errors Across Request Form Domains**
