# Supplementary material for: Assessment of histopathology and cytology request form documentation quality using six Sigma and Pareto analysis in Benghazi, Libya
Source: Diagn Pathol. 2025 Dec 17;21:6. doi: 10.1186/s13000-025-01740-0 (PMC12821826; doi:10.1186/s13000-025-01740-0)
Supplement: Supplementary file 2 — Supplementary Material 2 [file 13000_2025_1740_MOESM2_ESM.docx]

### ****Annex 1: Data Extraction Checklist for Histopathology and Cytology Request Form Documentation Quality****

**Study Title:** **Assessment of Histopathology and Cytology Request Form Documentation Quality Using Six Sigma and Pareto Analysis in Benghazi, Libya**
**Objective:** To systematically evaluate the completeness of documentation in histopathology and cytology request forms across five pre-analytical domains.

**Instructions for Data Extractors:**

1. For each request form, review each of the 15 indicators listed below.
2. Score each indicator as "Mentioned" or "Missing" based on the operational definitions provided.
3. **"Mentioned"**: The information is **clearly, completely, and legibly** recorded in the designated area of the request form.
4. **"Missing"**: The information is **absent, entirely illegible, or so incomplete** that it cannot be used (e.g., only a first name, an unspecified "mass," or an unreadable signature).

**Data Extraction Checklist**

| **Domain** | **Quality Indicator** | **Operational Definition** | **Score (Mentioned/Missing)** | **Notes** |
| --- | --- | --- | --- | --- |
| **A. Patient Identification** | **A1. Full Name** | Patient's name consisting of **at least three components** (e.g., first, middle, and family name; or first, father's, and family name). Two components or initials only are considered "missing." | ☐ Mentioned ☐ Missing |  |
|  | **A2. Date of Birth / Age** | Either the full date of birth (DD/MM/YYYY) or the patient's age in years. | ☐ Mentioned ☐ Missing |  |
|  | **A3. Gender** | The biological sex of the patient (male/female). | ☐ Mentioned ☐ Missing |  |
|  | **A4. Contact Information** | A current address **or** a functional telephone number. | ☐ Mentioned ☐ Missing |  |
| **B. Clinical Information** | **B1. Provisional Diagnosis/Suspicion** | A concise clinical diagnosis or description of the suspected condition (e.g., "rule out breast carcinoma," "suspicious thyroid nodule"). Non-specific terms like "mass" or "lesion" without context are "missing." | ☐ Mentioned ☐ Missing |  |
|  | **B2. Relevant Medical History** | Information on past or current medical conditions relevant to the current specimen (e.g., "Hx of HPV," "previous resection of melanoma," "on tamoxifen"). | ☐ Mentioned ☐ Missing |  |
| **C. Specimen Details** | **C1. Hospital/Clinic Name** | The name of the referring facility or clinic where the specimen was obtained. | ☐ Mentioned ☐ Missing |  |
|  | **C2. Type of Specimen** | The anatomical nature of the specimen (e.g., "skin ellipse," "endometrial curettage," "lymph node," "breast biopsy"). | ☐ Mentioned ☐ Missing |  |
|  | **C3. Anatomical Site of Origin** | The precise location in the body from which the specimen was taken (e.g., "right breast, upper outer quadrant," "sigmoid colon," "neck, level II lymph node"). | ☐ Mentioned ☐ Missing |  |
|  | **C4. Date & Time of Collection** | The date **and** (if available) the time when the specimen was procured from the patient. | ☐ Mentioned ☐ Missing |  |
| **D. Requesting Clinician Information** | **D1. Clinician's Name** | The full name of the physician who requested the examination. | ☐ Mentioned ☐ Missing |  |
|  | **D2. Clinician's Specialty** | The medical specialty of the requesting clinician (e.g., "General Surgery," "Gynecology," "Gastroenterology"). | ☐ Mentioned ☐ Missing |  |
|  | **D3. Clinician's Contact Information** | A direct phone number, pager number, or email address for follow-up. | ☐ Mentioned ☐ Missing |  |
|  | **D4. Clinician's Signature** | A legible signature or a verifiable electronic signature. An unsigned stamped name is "Missing." | ☐ Mentioned ☐ Missing |  |
| **E. Ancillary Investigations** | **E1. Request for Special Stains / IHC / Molecular Tests** | Any specific request for tests beyond routine H&E staining (e.g., "request ZN stain for AFB," "perform HER2 IHC," "do BRAF mutation analysis"). A pre-printed list on the form that is not checked is "Missing." | ☐ Mentioned ☐ Missing |  |
